# Effect of proximal box elevation on fracture resistance and microleakage of premolars restored with ceramic endocrowns

**DOI:** 10.1371/journal.pone.0252269

**Published:** 2021-05-26

**Authors:** Hong Zhang, He Li, Qian Cong, Zhimin Zhang, Aobo Du, Ying Wang

**Affiliations:** 1 Hospital of Stomatology, Jilin University, Changchun, P.R China; 2 Key Laboratory of Bionic Engineering, Ministry of Education, Jilin University, Changchun, P.R China; Danube Private University, AUSTRIA

## Abstract

**Background:**

Restoration of endodontically treated premolar is in high risk for biomechanical failure, and often presents with subgingival margins. Proximal box elevation (PBE) has been used to relocate subgingival cavity outlines.

**Objective:**

To evaluate the influence of PBE on fracture resistance and gingival microleakage of premolars with endodontic access cavities following ceramic endocrown.

**Methods:**

Eighty sound maxillary premolars with standardized Class II cavities on mesial surfaces were randomly assigned to four groups (n = 20 in each group). Groups E1, E2 and E3, with proximal margins located in dentin/cementum, 2 mm below the cemento-enamel junction (CEJ), simulated subgingival location. Group E4 (supragingival group), with proximal margins located in enamel, 1 mm above the CEJ, was used as the positive control. For margin elevation of the proximal cavities, bulk-fill Smart Dentin Replacement (SDR), a visible light cured resin composite, was applied in group E1, and conventional resin composite (3M Z350 XT, a light-activated composite) was placed in group E2. Group E3 was only treated with a ceramic crown and served as the negative control. In all groups, computer-aided design (CAD) ceramic endocrowns were adhesively inserted, and fracture resistance, failure mode and microleakage were evaluated.

**Results:**

A higher fracture resistance value was observed in PBE groups E1 and E2, regardless of the materials used (P = 0.038, and 0.010, respectively, vs E3), and fracture resistance in group E1 was higher than that in group E2. In teeth without PBE, the percentage of catastrophic failures reached 70%. Compared to group E3, a lower frequency distribution of microleakage was detected in supragingival group E4 (P = 0.031). No increased percentage of microleakage was observed in groups treated with PBE.

**Conclusion:**

For endodontically treated maxillary premolars restored with ceramic endocrowns, PBE increases fracture resistance but not microleakage.

## Introduction

Because of the high risk of biomechanical failure, restoration of endodontically treated premolars remains a challenge in prosthetic and restorative dentistry [1]. Teeth may fracture due to the decrease of water content, and the absence of structural integrity associated with deep dental caries, trauma, or restorative and endodontic procedures [2, 3]. Additionally, endodontically treated teeth with deep proximal defects display maximal tooth fragility [4]. To minimize risk of fracture, premolar under endodontic therapy is suggested to follow full-coverage restorations [5].

Computer-aided design/computer-aided manufacturing (CAD/CAM) ceramic endocrown with minimally invasive preparations has been used for restoring endodontically treated teeth rather than post-core techniques [6]. Fiber post placement is efficacious to reduce failures of postendodontic restorations only with teeth that exhibit no coronal walls. Post insertion for teeth showing a minor substance loss should be critically reconsidered [7, 8]. However, because proximal defects are often located beneath gingiva, it is difficult for impression taking, rubber dam application, and adhesive cementation [9, 10]. Dry condition during cementation is also required, but marginal microleakage is hard to control [11]. A less invasive method to set cavity margins supragingivally was brought up by Dietschi et al. (1998) using a composite layer covered with an indirect ceramic restoration. This approach is commonly referred to as proximal box elevation (PBE) which is to set an intra-crevicular to a supragingival level by a proper resin composite onto the existing margin, similar mechanism like deep margin elevation [12–15]. After resetting the cavity margins to the supragingival level, a rubber dam is applied to maintain a dry working field, thus significantly facilitating PBE physical applications [16].

Polymerization of the composite layer is often accompanied by substantial volumetric shrinkage. This shrinkage may cause marginal leakage, secondary caries, cuspal deflection, which may be associated with microleakage and marginal staining at the tooth/restoration interface, leading to adhesion failure [17–20]. Previous studies suggested that a resin composite with bulk-fill technique exhibits several advantageous properties such as low polymerization shrinkage, low microleakage, good color stability, and low water sorption and solubility [21]. A few data demonstrated how a resin composite layer for PBE impacts fracture and marginal microleakage of root canal treated premolar with indirect restorations [22–25]. So, based on previous findings, we hypothesized that PBE technique is feasible following ceramic endocrown. However, how PBE with a resin composite impact the marginal quality and fracture behavior of premolars restored with CAD/CAM endocrowns remains unclear. In this study, we aimed to explore the effects of PBE technique on fracture resistance, marginal microleakage in premolars with CAD/CAM endocrowns, and to evaluate the difference of two kinds of materials in the relocation of the cervical margin.

## Materials and methods

### Specimen collection and preparation

**Fig 1:**
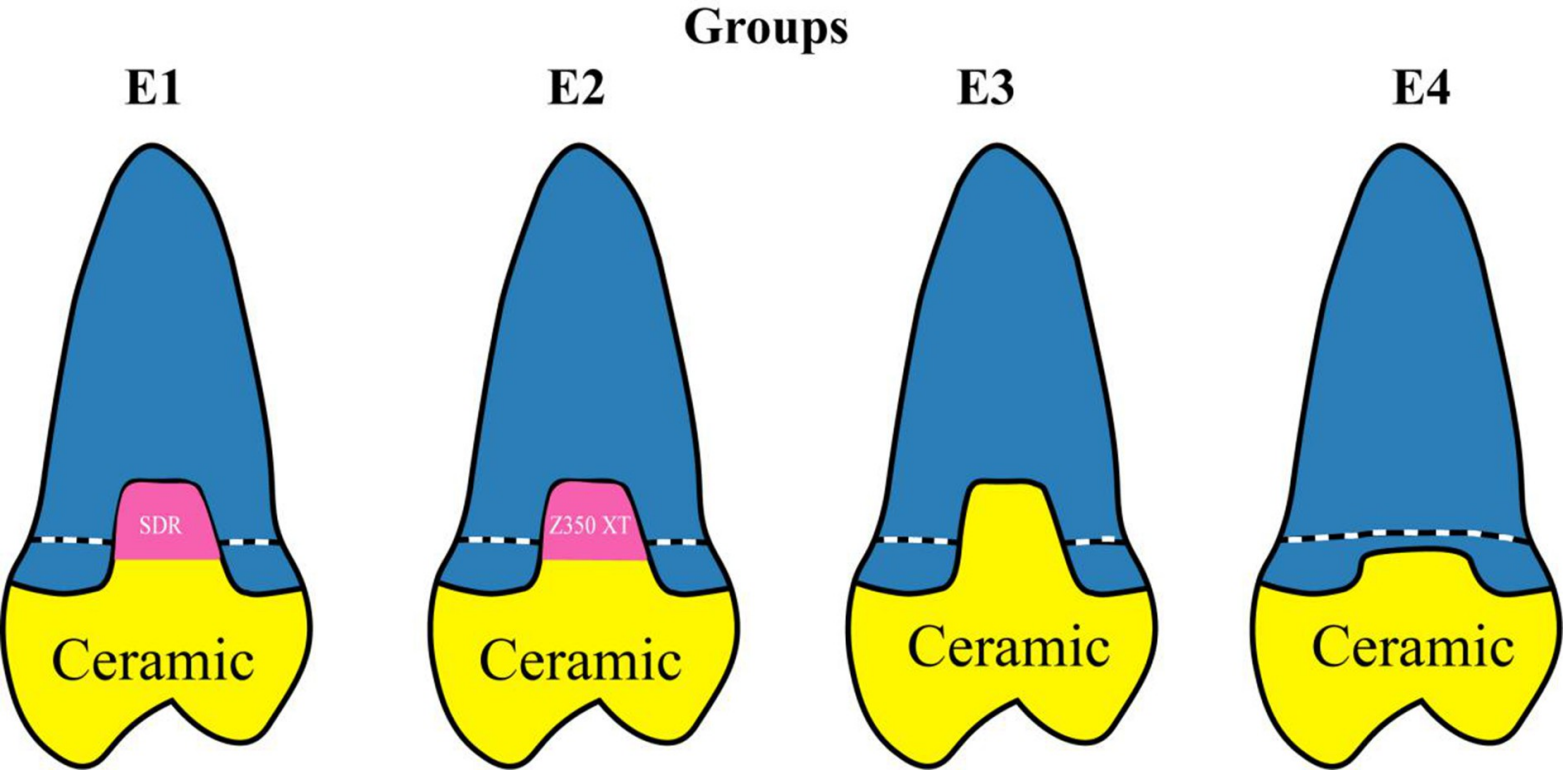
Restoration designs used in this study. Groups E1, E2 and E3, with proximal margins located in dentin/cementum, subgingival. Group E4 (supragingival group), with proximal margins located in enamel above the CEJ, as the positive control. Group E1 was with bulk-fill Smart Dentin Replacement, and E2 with conventional resin composite. Group E3 was treated with a ceramic crown, as the negative control.

This study was approved by the Ethics Committee of Jilin University, China on September 3rd, 2016 (No.2016-025). Signed informed consent was also obtained from all adult patients who needed teeth extraction. Eighty complete and caries-free human premolars were immersed in 0.1% thymol solution (Sigma-Aldrich, St. Louis, MO, USA) after extraction, and used in this *in vitro* study. After cleaning, the premolars were randomly allocated to four groups (n = 20 in each group). Root canal preparation was done with rotary instruments ProTaper Next (Dentsply Maillefer, Ballaigues, Switzerland) until the apical size reached ISO 30. The root canals were vertically filled with condensed gutta-percha (BeeFill, VDW, München, Germany) and an epoxy sealer (AH-Plus, DentsplyDe Trey, Konstanz, Germany). Standardized mesial-occlusal (MO) Class II cavities were designed as follows: 5 mm in buccal-lingual extension, 2 mm width at the cervical area. In groups E1, E2 and E3, all cervical margins were set 2 mm below cemento-enamel junction (CEJ), and 3 mm below CEJ in group E4. A 3 mm thick bulk-fill SDR flowable composite (Dentsply Caulk, Milford, DE, USA) was applied in group E1, and 1.5 mm thick Filtek Z350 XT conventional resin composite (3 M ESPE, St Paul, MN, USA) was used in group E2. At the same time, two increments were used to simulate PBE with a periodontal probe (Kang Qiao, Alton (Shanghai) Medical Instruments, Shanghai, China) in both group E1 and E2. In group E3, all cervical margins were left untreated. In group E4, enamel margins were treated with 37% phosphoric acid (Monobond Plus, Ivoclar Vivadent, Schaan, Liechtenstein) for 30 s, rinsed with water for 30 s, and then gently dried with air. Immediate dentin sealing (IDS) techniques were applied, and a Tetric N-Bond self-etching adhesive resin (Monobond Plus, Ivoclar Vivadent, Schaan, Liechtenstein) was applied to the dentin surface and left in place for 20 s. Excess solvent was removed by air-drying for 3 S. The bonding, together with a resin composite, was light cured using an LED polymerization device (SmartLite Focus, Dentsply Sirona, Ballaigues, Switzerland) at 1,200 mW/cm^2^ for 40 s. After placing the proximal composite layers, the cervical boxes of the MO-cavities were set 1 mm above CEJ in groups E1 and E2. The shape of cavities and composite increments of all groups are shown in Fig 1.

The endocrowns were designed and machined with the Cerec CAD/CAM system (D4DTechnologies, Richardson, TX, USA), showing all contact points and articulation for correct dynamic occlusion. The endocrown preparation consisted of deepening the central retention cavity, and extending 5 mm in depth from the occlusal floor with round internal line angles. A 2 mm reduction of the buccal and lingual cusps was done on all teeth. Internally, a flowable material (Filtek TM Supreme XTE Flowable; 3M ESPE, St. Paul, MN, USA) was used to seal the root canal and plan the chamber wall. A smooth chamber wall and divergent axial walls were preserved 8 degree towards the occlusal surface. The endocrown was gently positioned with finger pressure.

All cavity walls were polished, and sharp inner corners were rounded. Ceramic endocrowns were made using the CEREC 3D CAD-CAM unit (Sirona Dental Systems, Bensheim, Germany) and produced from lithium disilicate reinforced ceramic (IPS e.max CAD; Ivoclar Vivadent, Schaan, Liechtenstein). After adjustment, the internal surface of the restoration was rinsed with 96% ethanol (Tianyu, Jilin, China) and air-dried. To improve the strength, all the surfaces were etched with 5% hydrofluoric acid (Ivoclar Vivadent, Schaan, Liechtenstein) for 1 min, and then washed with air/water for 30 s. Silane (Ivoclar Vivaddent, Schaan, Liechtenstein) was used for 1 min and air dried. Before cementation, the resin composites for PBE in groups E1 and E2 were pre-treated with air abrasion (CoJet, 30 mm; 3M ESPE Platz, Seefeld, Germany) for 5 s, followed by an extensive clean with water spray. 37% phosphoric acid (Ivoclar Vivadent, Schaan, Liechtenstein) was spread on the enamel for 30 s and dentin for 15 s. The resin composites in groups E1 and E2 were then silanized (Monobond-S, IvoclarVivadent, Schaan, Liechtenstein), and dried without air-blow. Then the bonding system (Tetric N-Bond, Ivoclar Vivadent, Schaan, Liechtenstein) was used as described above for all groups. All endocrowns were luted with dual-cured Variolink II luting resin composite cement (Ivoclar Vivadent, Schaan, Liechtenstein), and then cemented. The surface was finished and polished with disks (Sof-Lex Pop-On; 3M ESPE, St. Paul, MN, USA) in decreasing granulations.

### Fracture resistance testing

Samples in each group were tested for fracture resistance. The roots of selected samples were covered with a 0.3 mm gum resin layer (Impregum Penta Soft, 3M ESPE, Seefeld, Germany) to simulate a periodontal ligament, and subsequently embedded in self-curing acrylic resin (Shanghai Dental Material Company, shanghai, China). The restoration margins were set approximately 2 mm above the feigned bone level. All restored specimens were stored in distilled water in the dark at 37°C for 72 h. Repeated mechanical stress was applied to all specimens under a computer-controlled masticator (custom made; Jilin University, Jilin, China) for 1,200,000 cycles, using tungsten carbide spheres (5.0 mm radius of curvature) of 49 N at 1.7 Hz [26], and simulating approximately 5 years of clinical usage. Then, all samples were placed vertically to the long axis of the tooth for fixation in a universal testing machine (NTS Technology, Tokyo, Japan), to produce a static compression force onto the tooth by a 5 mm steel sphere. The crosshead speed was set at 0.05 mm/s until fracture occurred.

Fractures were divided into two categories: favorable failures were repairable fractures above the level of bone recreation, and unfavorable failures were catastrophic fractures below the level of bone simulation. The numbers and percentage of each fracture were recorded.

### Microleakage testing

After cementation of the crowns for 1 h, the remaining specimens were immersed in water at room temperature for 24 h before thermocycling. The teeth were thermocycled using 500 cycles from 5°C to 55°C with a dwell time for 30 s under a thermocycling device (TC-501F; Weier, Suzhou, China) [27]. After thermocycling, the teeth were then covered with two coats of nail varnish 1 mm beyond the margins of the crown and immersed in 0.55% methylene-blue dye solution (Sigma-Aldrich, St. Louis, MO, USA) for 24 h at room temperature, followed by tap water washing for 10 min. PBE with the endocrowns was embedded in acrylic resin. And the teeth were split mesiodistally using a diamond wheel saw (Model SYJ-150A; Shenyang Kejing Equipment Manufacturing, Shenyang, China) under water-cooling. Two surfaces from each tooth were evaluated for gingival microleakage under a stereomicroscope (SZ51/61; Olympus, Tokyo, Japan). Dye penetration measurement was started from gingival margins. An independent examiner scored, and another trained examiner confirmed observation. The cervical marginal microleakage was recorded based on the following criteria: score 0 = no dye penetration, score 1 = dye penetration limited to enamel, score 2 = dye penetration beyond CEJ but limited to 2/3 of the cervical wall length, score 3 = dye penetration beyond 2/3 of the cervical wall length but not to the pulpal wall, and score 4 = dye penetration to the pulpal wall [10].

### Statistical analysis

SAS version 9.2 (SAS Institute, Cary, NC, USA) was used for data analysis, and data were expressed as mean ± standard deviation (SD). For fracture resistance testing, a one-way analysis of variance (ANOVA) and Bonferroni post hoc multiple comparison tests were used. Failure mode of four groups was analyzed by Chi–Square test using a software program (SPSS version 19.0; IBM, Armonk, NY, USA). For microleakage testing, a Kruskal-Wallis (K-W) test was used to assess differences among groups. Dwass-Steel-Critchlow-Fligner test was used as post hoc to investigate pairwise differences. The significant level was set at 0.05.

## Results

### Fracture resistance

The fracture resistance for four groups was obtained and shown in Table 1. Fracture resistance level was the highest in group E4, but it was not significantly different from group E1 (P = 0.059). Compared to group E3 (the negative control), fracture resistance level was increased significantly in groups E1 and E2 with PBE, and there was a significance between PBE and groups E1 and E2 (P = 0.010).

**Table 1:** Results of fracture resistance and fracture modes.

Groups	n	Fracture resistance (in Newton) means (±SD)	95% CI	Fracture mode	
				Repairable n (%)	Catastrophic n (%)
E1	20	1385.2 (±186.7)^a^	1251.7, 1518.7	14 (70)	6 (30)
E2	20	1154.1 (±311.2)^b^	931.4, 1376.8	12 (60)	8 (40)
E3	20	1083.6 (±397.1)^ab^	871.1, 1296.2	6 (30)	14 (70)
E4	20	1446.9 (±195.4)^a^	1307.2, 1586.7	18 (90)	2 (10)

**Note**: Values with same superscript letters in the same column were of no significant difference (P>0.05), those with different letters were of significant difference (P<0.05). Groups E1, E2 and E3, with proximal margins located in dentin/cementum, subgingival. Group E4 (supragingival group), with proximal margins located in enamel above the CEJ, as the positive control. Group E1 was with bulk-fill Smart Dentin Replacement, and E2 with conventional resin composite. Group E3 was treated with a ceramic crown, as the negative control. SD = Standard deviation, CI = confidence interval*, P = 0.010 compared with the positive control group E4. **, P = 0.020 compared with the positive control group E4. &, P = 0.070 compared with PBE group E2.

**Fig 2:**
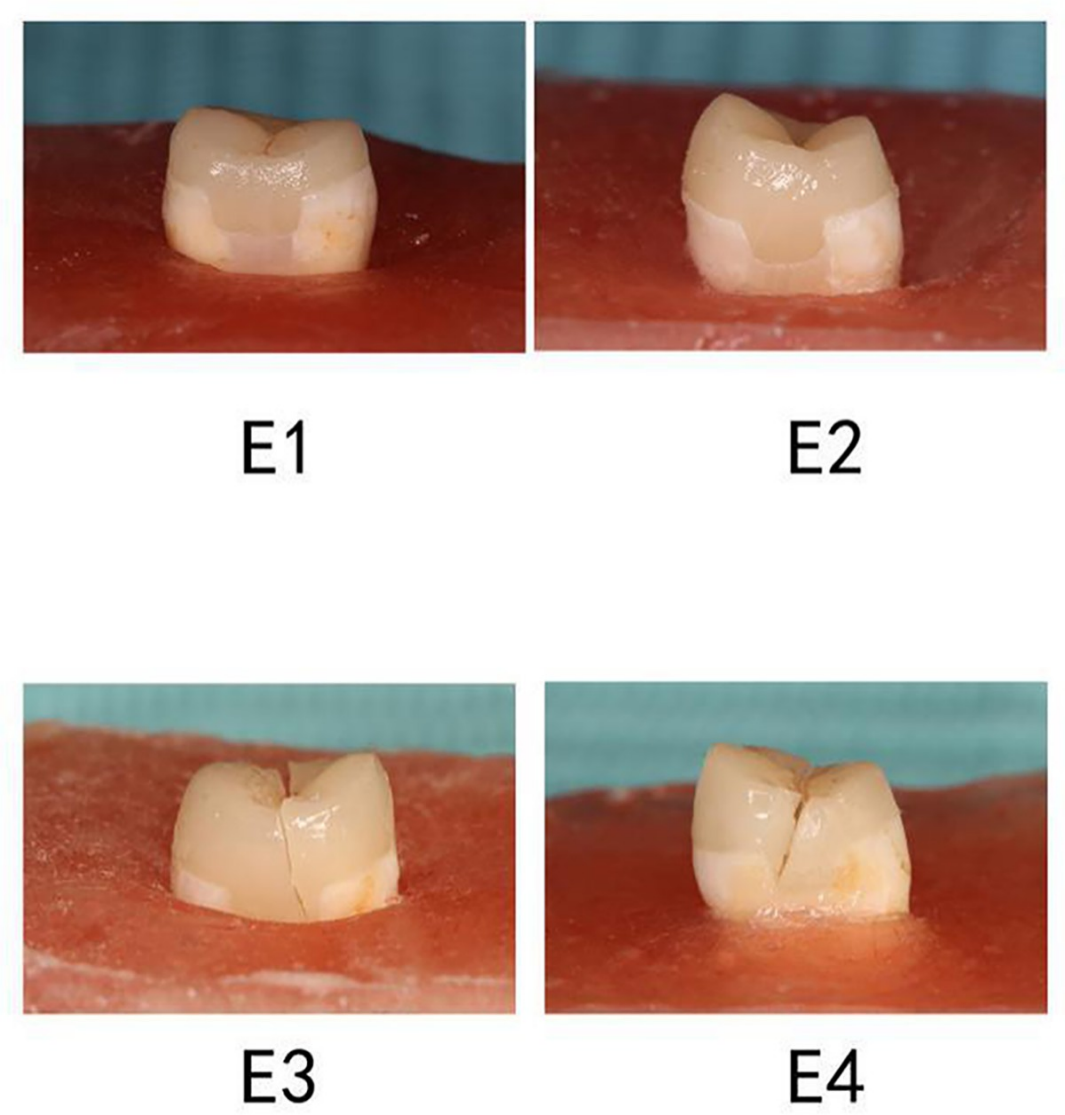
Images of fractures in different groups. Groups E1, E2 and E3, with proximal margins located in dentin/cementum, subgingival. Group E4 (supragingival group), with proximal margins located in enamel above the CEJ, as the positive control. Group E1 was with bulk-fill Smart Dentin Replacement, and E2 with conventional resin composite. Group E3 was treated with a ceramic crown, as the negative control.

Failure results are presented in Table 1 and Fig 2. It was suggested that, compared to group E3, resistance was increased and catastrophic fracture was decreased in groups E4 and E1 (both P = 0.020). Group E4 showed the highest resistance and least catastrophic fracture, followed by group E1 with bulk-fill SDR. However, there were no statistical significances between two filled groups E1 and E2 with PBE (P = 0.070) (Table 1). Representative failure types are also listed in Fig 2, showing the worst fracture in group E3 (the negative control), and the least fracture in group E4 (the positive control). Groups E1 and E2 with PBE fractured mainly at interface between dentin/composite layer or within resin composite for PBE, while groups E3 and E4 without PBE fractured from the surface to the interface through all layers vertically (Fig 2).

### Microleakage test results

**Fig 3:**
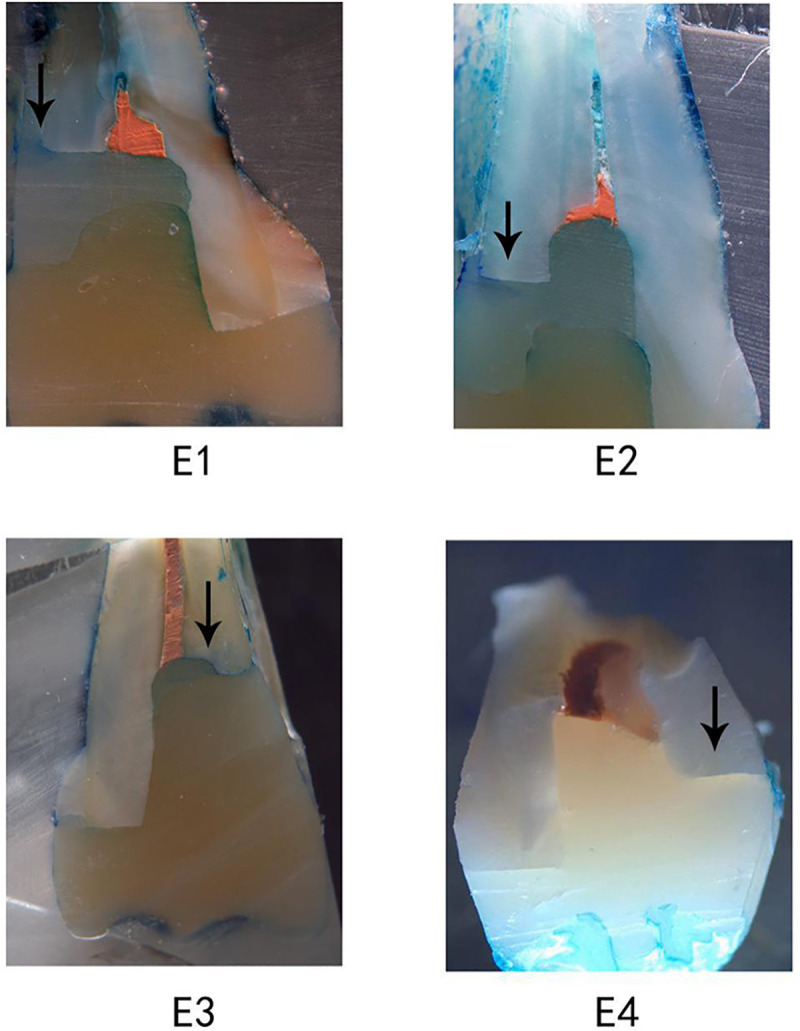
Representative stereomicroscopic photograph of microleakage in different groups. Groups E1, E2 and E3, with proximal margins located in dentin/cementum, subgingival. Group E4 (supragingival group), with proximal margins located in enamel above the CEJ, as the positive control. Group E1 was with bulk-fill Smart Dentin Replacement, and E2 with conventional resin composite. Group E3 was treated with a ceramic crown, as the negative control. Magnification used for observation of the specimens under stereomicroscope was 10×.

Microleakage in dentin/cementum margins is presented in Table 2, and representative stereomicroscopic photographs of microleakage in groups E1 to E4 are shown in Fig 3. The highest frequency distribution of score 0 and 1 was detected in the supragingival group E4 (60%), while the highest percentage of score 4 (12%) was found in group E3 (P = 0.026). The positive control group E4 also has the lowest percentage of score 1 (25%, P = 0.010), while group E1 presented a slightly higher frequency of score 2 (P = 0.078).

**Table 2:** Frequency distributions of microleakage scores on dentin/cementum margins.

Groups	Score 0	Score 1	Score 2	Score 3	Score 4
E1	3 (15%)	10 (50%)	4 (20%)	2 (10%)	1 (5%)
E2	2 (10%)	11 (55%)	3 (15%)	3 (15%)	1 (5%)
E3	1 (5%)	13 (65%)	2 (10%)	2 (10%)	2 (10%) *
E4	12 (60%) *	5 (25%) *	2 (10%)	1 (5%)	0 (0%)

Groups E1, E2 and E3, with proximal margins located in dentin/cementum, subgingival. Group E4 (supragingival group), with proximal margins located in enamel above the CEJ, as the positive control. Group E1 was with bulk-fill Smart Dentin Replacement, and E2 with conventional resin composite. Group E3 was treated with a ceramic crown, as the negative control. Group E4 had the highest percentage of score 0, while group E3 had the highest percentage for score 4.

*, P<0.050 compared with other groups.

## Discussion

With the development of material technology and clinical techniques, dental restorative treatment of large and deep cavities of posterior teeth has become feasible [28]. This study evaluated the fracture resistance and microleakage of premolars restored with ceramic endocrowns using PBE, and suggested that PBE significantly increases fracture resistance of the premolars, resulting in a more favorable fracture around the junction between the tooth and the material for PBE. A benefit of the PBE technique is that IDS technique can be performed concomitantly. This combined procedure increases retention, decreases marginal leakage, and has better bond strengths [29]. Previous studies have shown that PBE technique significantly increases the resistance of the restoration to fracture under a compressive load [30, 31]. Our study also revealed that PBE has a higher fracture load regardless of the materials used. The occurrence of high fracture resistance is most likely due to the improvements of the structural support [32]. The fracture patterns of the restored teeth are related to the brittle nature of ceramic. Generally, fracture line below bone level is considered as a non-restorable failure mode, and PBE affects the ratio of irreparable fractures to repairable fractures [33]. In line with previously published reports [33], our findings showed that majority of fracture pattern is vertical fracture from the occlusal fissure, which split the restoration and is located within the restoration or material for PBE. These findings can be explained by the layer of material for PBE, because the material for PBE and endocrown may function as a cohesive unit. Therefore, stress is distributed and PBE groups mainly display restorable failure modes. For ceramic restorations without PBE, fractures happen in the teeth when the force is dispersed through the small cervical structure, and the stress concentrates at the weakest area-the ceramic restoration and tooth interface. The failure mode of two PBE groups is similar, suggesting that the fracture mode depends on the construction in the cervical zone and cuspal inclination [34, 35], rather than the material for PBE.

Microleakage is a major reason of restoration failure [36]. So far, no restorative technique eliminates microleakage in gingival walls. It was reported that no difference was discovered in margin quality of ceramic restorations in dentin regardless of PBE [9, 10, 37]. Also, in this study, all testing groups showed some level of microleakage in gingival wall. Compared to E4 group, higher microleakage scores (score 1) were recorded in E3 group. A significant percentage of score 0 (no dye penetration) was found in supragingival group, suggesting the lowest microleakage in supragingival group. To reduce microleakage, new resin monomers with low polymerization shrinkage are suggested. Both bulk-fill SDR and traditional resin composite were tested, and no significant difference was found between them in this study, which are in accordance with the previous data [38–41]. This may be due to the fact that high molecular weight and the conformational flexibility impart optimizes flexibility. Lower dye penetration was reported for bulk-fill SDR [42]. The possible reason is that the thermal expansion coefficient of bulk-fill SDR is closer to the tooth tissue, so the microleakage is less in dentin margins of the cavity after temperature circulation [43].

The limitation of this study is the use of microleakage a two-dimensional measurement, and these results are most likely materials specific. Further study covering both cariologic and periodontal aspects, and clinical durability of PBE as a two-step restoration technique with larger sample size are clearly warranted.

## Conclusion

In conclusion, our study would suggest that premolars with gross lesions should be subsequently restored with full covering (CAD/CAM) ceramic restorations, while PBE seems to increase fracture resistance of ceramic endocrowns, and is able to decrease marginal microleakage of restorations showing gingival walls below the CEJ.

## Supporting information

S1 File (PDF)
