# Correction: Effect of proximal box elevation on fracture resistance and microleakage of premolars restored with ceramic endocrowns

**DOI:** 10.1371/journal.pone.0258038

**Published:** 2021-09-23

**Authors:** Hong Zhang, He Li, Qian Cong, Zhimin Zhang, Aobo Du, Ying Wang

The corresponding author’s email is incorrect. The correct email address is: zhangzm2019@yandex.com. The publisher apologizes for the error.

In the Specimen collection and preparation subsection of the Materials and methods, there is an error in the seventh sentence of the first paragraph. The correct sentence is: In groups E1, E2 and E3, all cervical margins were set 2 mm below cemento-enamel junction (CEJ), and 1 mm below CEJ in group E4.

In the Specimen collection and preparation subsection of the Materials and methods, there is an error in the eighth sentence of the first paragraph. The correct sentence is: A 3 mm thick bulk-fill SDR flowable composite (Dentsply Caulk, Milford, DE, USA) was applied in group E1, and 1.5 mm thick Filtek Z350 XT conventional resin composite (3 M ESPE, St Paul, MN, USA) was used in group E2, with two increments used to simulate PBE with a periodontal probe (Kang Qiao, Alton (Shanghai) Medical Instruments, Shanghai, China).

Additionally, the Specimen collection and preparation subsection of the Materials and methods incorrectly make reference to “Cerec CAD/CAM system (D4DTechnologies, Richardson, TX, USA)” and “CEREC 3D CAD-CAM unit (Sirona Dental Systems, Bensheim, Germany)”. These systems and commercial organizations were included in error and should have been omitted.

In the Fracture resistance subsection of the Results, there is an error in the third sentence of the first paragraph. The correct sentence is: Compared to group E3 (the negative control), fracture resistance level was increased significantly in groups E1 and E2 with PBE, and there was a significance between groups E1 and E2 (P = 0.010).

The Table 1 legend contains an error; the symbols * and ** were not utilized in Table 1. Please see the complete, correct Table 1 caption here.

**Table 1:** Results of fracture resistance and fracture modes.

Groups	n	Fracture resistance (in Newton) means(±SD)	95% CI	Fracture mode	
				Repairable n (%)	Catastrophic n (%)
E1	20	1385.2 (±186.7)^a^	1251.7, 1518.7	14 (70)	6 (30)
E2	20	1154.1 (±311.2)^b^	931.4, 1376.8	12 (60)	8 (40)
E3	20	1083.6 (±397.1)^ab^	871.1, 1296.2	6 (30)	14 (70)
E4	20	1446.9 (±195.4)^a^	1307.2, 1586.7	18 (90)	2 (10)

**Note**: Values with same superscript letters in the same column were of no significant difference (P>0.05), those with different letters were of significant difference (P<0.05). Groups E1, E2 and E3, with proximal margins located in dentin/cementum, subgingival. Group E4 (supragingival group), with proximal margins located in enamel above the CEJ, as the positive control. Group E1 was with bulk-fill Smart Dentin Replacement, and E2 with conventional resin composite. Group E3 was treated with a ceramic crown, as the negative control. SD = Standard deviation, CI = confidence interval.
