# Supplementary material for: Effect of proximal box elevation on fracture resistance and microleakage of premolars restored with ceramic endocrowns
Source: PLoS One. 2021 May 26;16(5):e0252269. doi: 10.1371/journal.pone.0252269 (PMC8153463; doi:10.1371/journal.pone.0252269)
Supplement: S1 File — (PDF) [file pone.0252269.s001.pdf]

CHAPTER 20

# Sample size and power calculations

## 20.1 Choices in the design of data collection

Multilevel modeling is typically motivated by features in existing data or the object of study—for example, voters classified by demography and geography, students in schools, multiple measurements on individuals, and so on. Consider all the examples in Part 2 of this book. In some settings, however, multilevel data structures arise by choice from the data collection process. We briefly discuss some of these options here.

### *Unit sampling or cluster sampling*

In a sample survey, data are collected on a set of units in order to learn about a larger population. In unit sampling, the units are selected directly from the population. In cluster sampling, the population is divided into clusters: first a sample of clusters is selected, then data are collected from each of the sampled clusters.

In *one-stage* cluster sampling, complete information is collected within each sampled cluster. For example, a set of classrooms is selected at random from a larger population, and then all the students within each sampled classroom are interviewed. In *two-stage* cluster sampling, a sample is performed within each sampled cluster. For example, a set of classrooms is selected, and then a random sample of ten students within each classroom is selected and interviewed. More complicated sampling designs are possible along these lines, including adaptive designs, stratified cluster sampling, sampling with probability proportional to size, and various combinations and elaborations of these.

### *Observational studies or experiments with unit-level or group-level treatments*

Treatments can be applied (or can be conceptualized as being applied in the case of a purely observational study) at individual or group levels; for example:

- In a medical study, different treatments might be applied to different patients, with patients clustered within hospitals that could be associated with varying intercepts or slopes.
- As discussed in Section 9.3, the Electric Company television show was viewed by classes, not individual students.
- As discussed in Section 11.2, child support enforcement policies are set by states and cities, not individuals.
- In the radon study described in Chapter 12, we can compare houses with and without basements within a county, but we can only study uranium as it varies between counties.

We present a longer list of such designs in the context of experiments in Section 22.4.

Typically, coefficients for factors measured at the individual level can be estimated more accurately than for group-level factors because there will be more individuals than groups; so $1/\sqrt{n}$ is more effective than $1/\sqrt{J}$ at reducing the standard error.

*Meta-analysis*

The sample size of a study can be increased in several ways:

- Gathering more data of the sort already in the study,
- Including more observations either in a nonclustered setting, as new observations in existing clusters, or new observations in new clusters
- Finding other studies performed under comparable (but not identical) conditions (so new observations in effect are like observations from a new "group").
- Finding other studies on related phenomena (again new observations from a different "group").

For example, in the study of teenage smoking in Section 11.3, these four options could be: (a) surveying more Australian adolescents about their smoking behavior, (b) taking more frequent measurements (for example, asking about smoking behavior every three months instead of every six months), (c) performing a similar survey in other cities or countries, or (d) performing similar studies of other unhealthy behaviors.

The first option is most straightforward—increasing $n$ decreases standard errors in proportion to $1/\sqrt{n}$. The others involve various sorts of multilevel models and are made more effective by collecting appropriate predictors at the individual and group levels. (As discussed in Section 12.3, the more that the variation is explained by external predictors, the more effective the partial pooling will be.) A challenge of multilevel design is to assess the effectiveness of these various strategies for increasing sample size. Finding data from other studies is often more feasible than increasing $n$ in an existing study, but then it is important to either find other studies that are similar, or to be able to model these differences.

*Sample size, design, and interactions*

Sample size is never large enough. As $n$ increases, we estimate more interactions, which typically are smaller and have relatively larger standard errors than main effects (for example, see the fitted regression on page 63 of log earnings on sex, standardized height, and their interaction). Estimating interactions is similar to comparing coefficients estimated from subsets of the data (for example, the coefficient for height among men, compared to the coefficient among women), thus reducing power because the sample size for each subset is halved, and also the differences themselves may be small. As more data are included in an analysis, it becomes possible to estimate these interactions (or, using multilevel modeling, to include them and partially pool them as appropriate), so this is not a problem. We are just emphasizing that, just as you never have enough money, because perceived needs increase with resources, your inferential needs will increase with your sample size.

## 20.2 Classical power calculations: general principles, as illustrated by estimates of proportions

Questions of data collection can typically be expressed in terms of estimates and standard errors for quantities of interest. This chapter follows the usual focus on estimating population averages, proportions, and comparisons in sample surveys; or estimating treatment effects in experiments and observational studies. However, the general principles apply for other inferential goals such as prediction and data reduction. The paradigmatic problem of power calculation is the estimation of a parameter $\theta$ (for example, a regression coefficient such as would arise in estimating a difference or treatment effect), with the sample size determining the standard error.

*Effect sizes and sample sizes*

In designing a study to maximize the power of detecting a statistically significant comparison, it is generally better, if possible, to double the effect size $\theta$ than to double the sample size $n$, since standard errors of estimation decrease with the square root of the sample size. This is one reason, for example, why potential toxins are tested on animals at many times their exposure levels in humans; see Exercise 20.3.

Studies are designed in several ways to maximize effect size:

- In drug studies, setting doses as low as ethically possible in the control group and as high as ethically possible in the experimental group.
- To the extent possible, choosing individuals that are likely to respond strongly to the treatment. For example, the Electric Company experiment described in Section 9.3 was performed on poorly performing classes in each grade, for which it was felt there was more room for improvement.

In practice, this advice cannot be followed completely. In the social sciences, it can be difficult to find an intervention with *any* noticeable positive effect, let alone to design one where the effect would be doubled. Also, when treatments in an experiment are set to extreme values, generalizations to more realistic levels can be suspect; in addition, missing data in the control group may be more of a problem if the control treatment is ineffective. Further, treatment effects discovered on a sensitive subgroup may not generalize to the entire population. But, on the whole, conclusive effects on a subgroup are generally preferred to inconclusive but more generalizable results, and so conditions are usually set up to make effects as large as possible.

*Power calculations*

Before data are collected, it can be useful to estimate the precision of inferences that one expects to achieve with a given sample size, or to estimate the sample size required to attain a certain precision. This goal is typically set in one of two ways:

- Specifying the standard error of a parameter or quantity to be estimated, or
- Specifying the probability that a particular estimate will be "statistically significant," which typically is equivalent to ensuring that its confidence interval will exclude the null value.

In either case, the sample size calculation requires assumptions that typically cannot really be tested until the data have been collected. Sample size calculations are thus inherently hypothetical.

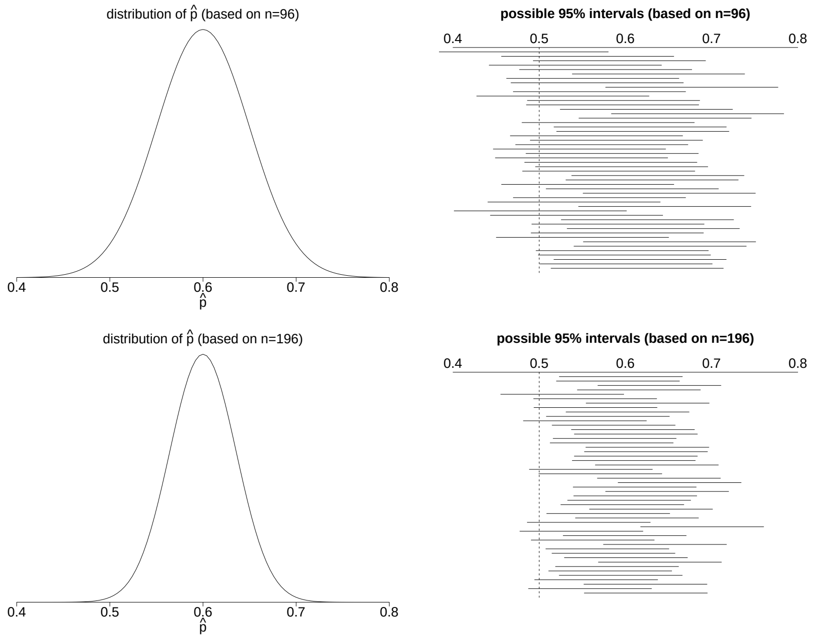


Figure 20.1 *Illustration of simple sample size calculations.*
*Top row: (a) distribution of the sample proportion $\hat{p}$ if the true population proportion is $p = 0.6$, based on a sample size of 96; (b) several possible 95% intervals for $p$ based on a sample size of 96. The power is 50%—that is, the probability is 50% that a randomly generated interval will be entirely to the right of the comparison point of 0.5.*
*Bottom row: corresponding graphs for a sample size of 196. Here the power is 80%.*

*Sample size to achieve a specified standard error*

To understand these two kinds of calculations, consider the simple example of estimating the proportion of the population who support the death penalty (under a particular question wording). Suppose we suspect the population proportion is around 60%. First, consider the goal of estimating the true proportion $p$ to an accuracy (that is, standard error) of no worse than 0.05, or 5 percentage points, from a simple random sample of size $n$. The standard error of the mean is $\sqrt{p(1-p)/n}$. Substituting the guessed value of 0.6 for $p$ yields a standard error of $\sqrt{0.6 \cdot 0.4/n} = 0.49/\sqrt{n}$, and so we need $0.49/\sqrt{n} \leq 0.05$, or $n \geq 96$. More generally, we do not know $p$, so we would use a conservative standard error of $\sqrt{0.5 \cdot 0.5/n} = 0.5/\sqrt{n}$, so that $0.5/\sqrt{n} \leq 0.05$, or $n \geq 100$.

*Sample size to achieve a specified probability of obtaining statistical significance*

Second, suppose we have the goal of demonstrating that more than half the population supports the death penalty—that is, that $p > 1/2$—based on the estimate $\hat{p} = y/n$ from a sample of size $n$. As above, we shall evaluate this under the hypothesis that the true proportion is $p = 0.60$, using the conservative standard error for $\hat{p}$ of $\sqrt{0.5 \cdot 0.5/n} = 0.5/\sqrt{n}$. The 95% confidence interval for $p$ is $[\hat{p} \pm 1.96 \cdot 0.5/\sqrt{n}]$, and classically we would say we have demonstrated that $p > 1/2$ if the interval lies entirely above 1/2; that is, if $\hat{p} > 0.5 + 1.96 \cdot 0.5/\sqrt{n}$. The estimate must be at least 1.96 standard errors away from the comparison point of 0.5.

A simple, but not quite correct, calculation, would set $\hat{p}$ to the hypothesized value

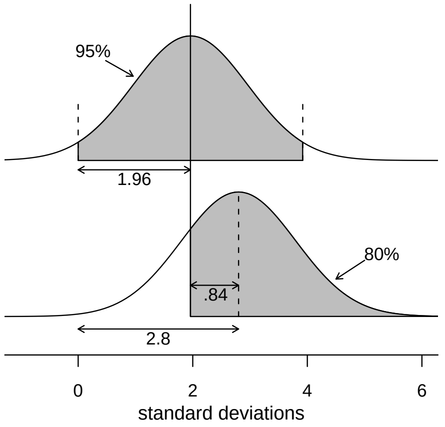


Figure 20.2 *Sketch illustrating that, to obtain 80% power for a 95% confidence interval, the true effect size must be at least 2.8 standard errors from zero (assuming a normal distribution for estimation error). The top curve shows that the estimate must be at least 1.96 standard errors from zero for the 95% interval to be entirely positive. The bottom curve shows the distribution of the parameter estimates that might occur, if the true effect size is 2.8. Under this assumption, there is an 80% probability that the estimate will exceed 1.96. The two curves together show that the lower curve must be centered all the way at 2.8 to get an 80% probability that the 95% interval will be entirely positive.*

of 0.6, so that the requirement is $0.6 > 0.5 + 1.96 \cdot 0.5/\sqrt{n}$, or $n > (1.96 \cdot 0.5/0.1)^2 = 96$. This is mistaken, however, because it confuses the assumption that $p = 0.6$ with the claim that $\hat{p} > 0.6$. In fact, if $p = 0.6$, then $\hat{p}$ depends on the sample, and it has an approximate normal distribution with mean 0.6 and standard deviation $\sqrt{0.6 \cdot 0.4/n} = 0.49/\sqrt{n}$; see Figure 20.1a.

To determine the appropriate sample size, we must specify the desired *power*—that is, the probability that a 95% interval will be entirely above the comparison point of 0.5. Under the assumption that $p = 0.6$, choosing $n = 96$ yields 50% power: there is a 50% chance that $\hat{p}$ will be more than 1.96 standard deviations away from 0.5, and thus a 50% chance that the 95% interval will be entirely greater than 0.5.

The conventional level of power in sample size calculations is 80%: we would like to choose $n$ such that 80% of the possible 95% confidence intervals will not include 0.5. When $n$ is increased, the estimate becomes closer (on average) to the true value, and the width of the confidence interval decreases. Both these effects (decreasing variability of the estimator and narrowing of the confidence interval) can be seen in going from the top half to the bottom half of Figure 20.1.

To find the value of $n$ such that exactly 80% of the estimates will be at least 1.96 standard errors from 0.5, we need

$$0.5 + 1.96\,\text{s.e.} = 0.6 - 0.84\,\text{s.e.}$$

Some algebra then yields $(1.96 + 0.84)\,\text{s.e.} = 0.1$. We can then substitute $\text{s.e.} = 0.5/\sqrt{n}$ and solve for $n$.

*2.8 standard errors from the comparison point*

In summary, to have 80% power, the true value of the parameter must be 2.8 standard errors away from the comparison point: the value 2.8 is 1.96 from the 95% interval, plus 0.84 to reach the $80^{th}$ percentile of the normal distribution. The

bottom row of Figure 20.1 illustrates: with $n = (2.8 \cdot 0.49/0.1)^2 = 196$, and if the true population proportion is $p = 0.6$, there is an 80% chance that the 95% confidence interval will be entirely greater than 0.5, thus conclusively demonstrating that more than half the people support the death penalty.

These calculations are only as good as their assumptions; in particular, one would generally not know the true value of $p$ before doing the study. Nonetheless, power analyses can be useful in giving a sense of the size of effects that one could reasonably expect to demonstrate with a study of given size. For example, a survey of size 196 has 80% power to demonstrate that $p > 0.5$ if the true value is 0.6, and it would easily detect the difference if the true value were 0.7; but if the true $p$ were equal to 0.56, say, then the difference would be only $0.06/(0.5/\sqrt{196}) = 1.6$ standard errors away from zero, and it would be likely that the 95% interval for $p$ would include 1/2, even in the presence of this true effect. Thus, if the primary goal of the survey were to conclusively detect a difference from 0.5, it would probably not be wise to use a sample of only $n = 196$ unless we suspect the true $p$ is at least 0.6. Such a small survey would "not have the power to" reliably detect differences of less than 0.1.

*Estimates of hypothesized proportions*

The standard error of a proportion $p$, if it is estimated from a sample of size $n$, is $\sqrt{p(1-p)/n}$, which has an upper bound of $0.5/\sqrt{n}$. This upper bound is very close to the actual standard error for a wide range of probabilities $p$ near 1/2: for example, for $\hat{p} = 0.5$, $\sqrt{0.5 \cdot 0.5} = 0.5$ exactly; for $\hat{p} = 0.6$ or 0.4, $\sqrt{0.6 \cdot 0.4} = 0.49$,; and for $\hat{p} = 0.7$ or 0.3, $\sqrt{0.7 \cdot 0.3} = 0.46$.

If the goal is a specified standard error, then a conservative required sample size is determined by s.e.$= 0.5/\sqrt{n}$, so that $n = (0.5/\text{s.e.})^2$ or, more precisely, $n = p(1-p)/(\text{s.e.})^2$, given a hypothesized $p$ near 0 or 1.

If the goal is 80% power to distinguish $p$ from a specified value $p_0$, then a conservative required sample size is $n = (2.8 \cdot 0.5/(p - p_0))^2$ or, more precisely, $n = p(1-p)(2.8/(p-p_0))^2$.

*Simple comparisons of proportions: equal sample sizes*

The standard error of a difference between two proportions is, by a simple probability calculation, $\sqrt{p_1(1-p_1)/n_1 + p_2(1-p_2)/n_2}$, which has an upper bound of $0.5\sqrt{1/n_1 + 1/n_2}$. If we make the restriction $n_1 = n_2 = n/2$ (equal sample sizes in the two groups), the upper bound on the standard error becomes simply $1/\sqrt{n}$. A specified standard error can then be attained with a sample size of $n = 1/(\text{s.e.})^2$.

If the goal is 80% power to distinguish between hypothesized proportions $p_1$ and $p_2$ with a study of size $n$, equally divided between the two groups, a conservative sample size is $n = [2.8/(p_1 - p_2)]^2$ or, more precisely, $n = 2[p_1(1-p_1) + p_2(1-p_2)] \cdot [2.8/(p_1 - p_2)]^2$.

For example, suppose we suspect that the death penalty is 10% more popular in the United States than in Canada, and we plan to conduct surveys in both countries on the topic. If the surveys are of equal sample size, $n/2$, how large must $n$ be so that there is an 80% chance of achieving statistical significance, if the true difference in proportions is 10%? The standard error of $\hat{p}_1 - \hat{p}_2$ is approximately $1/\sqrt{n}$, so for 10% to be 2.8 standard errors from zero, we must have $n > (2.8/0.10)^2 = 784$, or a survey of 392 persons in each country.

*Simple comparisons of proportions: unequal sample sizes*

In observational epidemiology, it is common to have unequal sample sizes in comparison groups. For example, consider a study in which 20% of units are "cases" and 80% are "controls."

First, consider the goal of estimating the difference between the treatment and control groups, to some specified precision. The standard error of the difference is $\sqrt{p_1(1-p_1)/(0.2n) + p_2(1-p_2)/(0.8n)}$, and this expression has an upper bound of $0.5\sqrt{1/(0.2n) + 1/(0.8n)} = 0.5\sqrt{1/(0.2) + 1/(0.8)}/\sqrt{n} = 1.25/\sqrt(n)$. A specified standard error can then be attained with a sample size of $n = (1.25/\text{s.e.})^2$.

Second, suppose we want have sufficient total sample size $n$ to achieve 80% power to detect a difference of 10%, again with 20% of the sample size in one group and 80% in the other. Again, the standard error of $\hat{p}_1 - \hat{p}_2$ is bounded by $1.25/\sqrt{n}$, so for 10% to be 2.8 standard errors from zero, we must have $n > (2.8 \cdot 1.25/0.10)^2 = 1225$, or 245 cases and 980 controls.

## 20.3 Classical power calculations for continuous outcomes

Sample size calculations proceed much the same way with continuous outcomes, with the added difficulty that the population standard deviation must also be specified along with the hypothesized effect size. We shall illustrate with a proposed experiment adding zinc to the diet of HIV-positive children in South Africa. In various other populations, zinc and other micronutrients have been found to reduce the occurrence of diarrhea, which is associated with immune system problems, as well as to slow the progress of HIV. We first consider the one-sample problem—how large a sample size would we expect to need to measure various outcomes to a specified precision—and then move to two-sample problems comparing treatment to control groups.

*Estimates of means*

Suppose we are trying to estimate a population mean value $\theta$ from data $y_1, \ldots, y_n$, a random sample of size $n$. The quick estimate of $\theta$ is the sample mean, $\bar{y}$, which has a standard error of $\sigma/\sqrt{n}$, where $\sigma$ is the standard deviation of $y$ in the population. So if the goal is to achieve a specified s.e. for $\bar{y}$, then the sample size must be at least $n = (\sigma/\text{s.e.})^2$.

If the goal is 80% power to distinguish $\theta$ from a specified value $\theta_0$, then a conservative required sample size is $n = (2.8\sigma/(\theta - \theta_0))^2$.

*Simple comparisons of means*

The standard error of $\bar{y}_1 - \bar{y}_2$ is $\sqrt{\sigma_1^2/n_1 + \sigma_2^2/n_2}$. If we make the restriction $n_1 = n_2 = n/2$ (equal sample sizes in the two groups), the standard error becomes simply s.e. $= \sqrt{2(\sigma_1^2 + \sigma_2^2)}/\sqrt{n}$. A specified standard error can then be attained with a sample size of $n = 2(\sigma_1^2 + \sigma_2^2)/(\text{s.e.})^2$. If we further suppose that the variation is the same within each of the groups ($\sigma_1 = \sigma_2 = \sigma$), then s.e. $= 2\sigma/\sqrt{n}$, and the required sample size is $n = (2\sigma/\text{s.e.})^2$.

If the goal is 80% power to detect a difference of $\Delta$, with a study of size $n$, equally divided between the two groups, then the required sample size is $n = 2(\sigma_1^2 + \sigma_2^2)(2.8/\Delta)^2$. If $\sigma_1 = \sigma_2 = \sigma$, this simplifies to $(5.6\sigma/\Delta)^2$.

For example, consider the effect of zinc supplements on young children's growth. Results of published studies suggest that zinc can improve growth by approximately

Rosado et al. (1997), Mexico

| Treatment | Sample size | Avg. # episodes in a year ± s.e. |
|---|---|---|
| placebo | 56 | $1.1 \pm 0.2$ |
| iron | 54 | $1.4 \pm 0.2$ |
| zinc | 54 | $0.7 \pm 0.1$ |
| zinc + iron | 55 | $0.8 \pm 0.1$ |

Ruel et al. (1997), Guatemala

| Treatment | Sample size | Avg. # episodes per 100 days [95% c.i.] |
|---|---|---|
| placebo | 44 | 8.1 [5.8, 10.2] |
| zinc | 45 | 6.3 [4.2, 8.9] |

Lira et al. (1998), Brazil

| Treatment | Sample size | % days with diarrhea | Prevalence ratio [95% c.i.] |
|---|---|---|---|
| placebo | 66 | 5% | 1 |
| 1 mg zinc | 68 | 5% | 1.0 [0.72, 1.4] |
| 5 mg zinc | 71 | 3% | 0.68 [0.49, 0.95] |

Muller et al. (2001), West Africa

| Treatment | Sample size | # days with diarrhea/ total # days |
|---|---|---|
| placebo | 329 | 997/49021 = 0.020 |
| zinc | 332 | 869/49086 = 0.018 |

Figure 20.3 *Results from various experiments studying the effects of zinc supplements on diarrhea in children. We use this information to hypothesize the effect size $\Delta$ and within-group standard deviation $\sigma$ for our planned experiment.*

0.5 standard deviations. That is, $\Delta = 0.5\sigma$ in the our notation. To have 80% power to detect an effect size, it would be sufficient to have a total sample size of $n = (5.6/0.5)^2 = 126$, or $n/2 = 63$ in each group.

*Estimating standard deviations using results from previous studies*

Sample size calculations for continuous outcomes are based on estimated effect sizes and standard deviations in the population—that is, $\Delta$ and $\sigma$. Guesses for these parameters can be estimated or deduced from previous studies. We illustrate with the design of a study to estimate the effects of zinc on diarrhea in children. Various experiments have been performed on this topic—Figure 20.3 summarizes the results, which we shall use to get a sense of the sample size required for our study.

We consider the studies reported in Figure 20.3 in order. For Rosado et al. (1997), we shall estimate the effect of zinc by averaging over the iron and no-iron cases, thus an estimated $\Delta$ of $\frac{1}{2}(1.1+1.4) - \frac{1}{2}(0.7+0.8) = 0.5$ episodes in a year, with a standard error of $\sqrt{\frac{1}{4}(0.2^2+0.2^2)+\frac{1}{4}(0.1^2+0.1^2)} = 0.15$. From this study, it would be reasonable to hypothesize that zinc reduces diarrhea in that population by an average of about 0.3 to 0.7 episodes per year. Next, we can deduce the within-group standard deviations $\sigma$ using the formula s.e.$= \sigma/\sqrt{n}$; thus the standard deviations are $0.2 \cdot \sqrt{56} = 1.5$ for the placebo group, and similarly for the other three groups are 1.5, 0.7, and 0.7, respectively. (Since the number of episodes is

bounded below by zero, it makes sense that when the mean level goes down, the standard deviation decreases also.)

Assuming an effect size of $\Delta = 0.5$ episodes per year and within-group standard deviations of 1.5 and 0.7 for the control and treatment groups, we can evaluate the power of a future study with $n/2$ children in each group. The estimated difference would have a standard error of $\sqrt{1.5^2/(n/2) + 0.7^2/(n/2)} = 2.4/\sqrt{n}$, and so for the effect size to be at least 2.8 standard errors away from zero (and thus to have 80% power to attain statistical significance), $n$ would have to be at least $(2.8 \cdot 2.4/0.5)^2 = 180$ persons in the two groups.

Now turning to the Ruel et al. (1997) study, we first see that rates of diarrhea—for control and treated children both—are much higher than in the previous study: 8 episodes per hundred days, which corresponds to 30 episodes per year, more than 20 times the rate in the earlier group. We are clearly dealing with much different populations here. In any case, we can divide the confidence interval widths by 4 to get standard errors—thus, 1.1 for the placebo group and 1.2 for the treated group—yielding an estimated treatment effect of 1.8 with standard error 1.6, which is consistent with a treatment effect of nearly zero or as high as about 4 episodes per 100 days. When compared to the average observed rate in the control group, the estimated treatment effect from this study is about half that of the Rosado et al. (1997) experiment: $1.8/8.1 = 0.22$, compared to $0.5/1.15 = 0.43$, which suggests a higher sample size might be required. However, the wide confidence bounds of the Ruel et al. (1997) study make it consistent with the larger effect size.

Next, Lira et al. (1998) report the average percent of days with diarrhea of children in the control and two treatment groups corresponding to a low (1 mg) or high (5 mg) dose of zinc. We shall consider only the 5 mg condition as this is closer to the treatment we are considering in our experiment. The estimated effect of the treatment is to multiply the number of days with diarrhea by 68%—that is, a reduction of 32%, which again is consistent with the approximate 40% decrease found in the first study. To make a power calculation, we first convert the confidence interval $[0.49, 0.95]$ for this multiplicative effect to the logarithmic scale—thus, an additive effect of $[-0.71, -0.05]$ on the logarithm—then divide by 4 to get an estimated standard error of 0.16 on this scale. The estimated effect of 0.68 is $-0.38$ on the log scale, thus 2.4 standard errors away from zero. For this effect size to be 2.8 standard errors from zero, we would need to increase the sample size by a factor of $(2.8/2.4)^2 = 1.4$, thus moving from approximately 70 children to approximately 100 in each of the two groups.

Finally, Muller et al. (2001) compare the proportion of days with diarrhea, which declined from 2.03% in the controls to 1.77% among children who received zinc. Unfortunately, no standard error is reported for this 13% decrease, and it is not possible to compute it from the information in the article. However, the estimates of within-group variation $\sigma$ from the other studies would lead us to conclude that we would need a very large sample size to be likely to reach statistical significance, if the true effect size were only 10%. For example, from the Lira et al. (1998) study, we estimate a sample size of 100 in each group is needed to detect an effect of 32%; thus to detect a true effect of 13% we would need a sample size of $100(0.32/0.13)^2 = 600$.

These calculations are necessarily speculative; for example, to detect an effect of 10% (instead of 13%), the required sample size would be $100(0.32/0.10)^2 = 1000$ per group, a huge change considering the very small change in hypothesized treatment effects. Thus, it would be misleading to think of these as "required sample sizes." Rather, these calculations tell us how large the effects are that we could expect to have a good chance of discovering, given any specified sample size.

The first two studies in Figure 20.3 report the frequency of episodes, whereas the last two studies give the proportion of days with diarrhea, which is proportional to the frequency of episodes multiplied by the average duration of each episode. Other data (not shown here) show no effect of zinc on average duration, and so we treat all four studies as estimating the effects on frequency of episodes.

In conclusion, a sample size of about 100 per treatment group should give adequate power to detect an effect of zinc on diarrhea, if its true effect is to reduce the frequency, on average, by 30%–50% compared to no treatment. A sample size of 200 per group would have the same power to detect effects a factor $\sqrt{2}$ smaller, that is, effects in the 20%–35% range.

*Including more regression predictors*

Now suppose we are comparing treatment and control groups with additional pre-treatment data available on the children (for example, age, height, weight, and health status at the start of the experiment). These can be included in a regression. For simplicity, we consider a model with no interactions—that is, with coefficients for the treatment indicator and the other inputs—in which case, the treatment coefficient represents the causal effect, the comparison between the two groups after controlling for pre-treatment differences.

Sample size calculations for this new study are exactly as before, except that the within-group standard deviation $\sigma$ is replaced by the residual standard deviation of the regression. This can be hypothesized in its own right or in terms of the added predictive power of the pre-treatment data. For example, if we hypothesize a within-group standard deviation of 0.2, then a residual standard deviation of 0.14 would imply that half the variance within any group is explained by the regression model, which would actually be pretty good.

Adding predictors tends to decrease the residual standard deviation and thus reduce the required sample size for any specified level of precision or power.

*Estimation of regression coefficients more generally*

More generally, sample sizes for regression coefficients and other estimands can be calculated using the rule that standard errors are proportional to $1/\sqrt{n}$; thus, if inferences exist under a current sample size, effect sizes can be estimated and standard errors extrapolated for other hypothetical samples.

We illustrate with the example of the survey earnings and height discussed in Chapter 4. The coefficient for the sex-earnings interaction in model (4.2) on page 63 is plausible (a positive interaction, implying that an extra inch of height is worth 0.7% more for men than for women), but it is not statistically significant—the standard error is 1.9%, yielding a 95% interval of $[-3.1, 4.5]$, which contains zero.

*Simple sample size and power calculations.* How large a sample size would be needed for the coefficient on the interaction to be statistically significant? A simple calculation uses the fact that standard errors are proportional to $1/\sqrt{n}$. For a point estimate of 0.7% to achieve statistical significance, it would need a standard error of 0.35%, which would require the sample size to be increased by a factor of $(1.9\%/0.35\%)^2 = 29$. The original survey had a sample of 1192; this implies a required sample size of $29 \cdot 1192 = 35{,}000$.

To extend this to a power calculation, we suppose that the true $\beta$ for the interaction is equal to 0.7% and that the standard error is as we have just calculated. With

a standard error of 0.35%, the estimate from the regression would then be statistically significant only if $\hat{\beta} > 0.7\%$ (or, strictly speaking, if $\hat{\beta} < -0.7\%$, but that latter possibility is highly unlikely given our assumptions). If the true coefficient is $\beta$, we would expect the estimate from the regression to possibly take on values in the range $\beta \pm 0.35\%$ (that is what is meant by "a standard error of 0.35%"), and thus if $\beta$ truly equals 0.7%, we would expect $\hat{\beta}$ to exceed 0.7%, and thus achieve statistical significance, with a probability of 1/2—that is, 50% power. To get 80% power, we need the true $\beta$ to be 2.8 standard errors from zero, so that there is an 80% probability that $\hat{\beta}$ is at least 2 standard errors from zero. If $\beta = 0.7\%$, then its standard error would have to be no greater than $0.7\%/2.8 = 0.25\%$, so that the survey would need a sample size of $(1.9\%/0.25\%)^2 \cdot 1192 = 70{,}000$.

This power calculation is only provisional, however, because it makes the very strong assumption that the $\beta$ is equal to 0.7%, the estimate that we happened to obtain from our survey. But the estimate from the regression is $0.7\% \pm 1.9\%$, which implies that these data are consistent with a low, zero, or even negative value of the true $\beta$ (or, in the other direction, a true value that is greater than the point estimate of 0.7%). If the true $\beta$ is actually less than 0.7%, then even a sample size of 70,000 will be insufficient for 80% power.

This is not to say the power calculation is useless but just to point out that, even when done correctly, it is based on an assumption that is inherently untestable from the available data (hence the need for a larger study). So we should not necessarily expect statistical significance from a proposed study, even if the sample size has been calculated correctly.

## 20.4 Multilevel power calculation for cluster sampling

With multilevel data structures and models, power calculations become more complicated because there is the option to set the sample size at each level. In a cluster sampling design, one can choose the number of clusters to sample and the number of units to sample within each cluster. In a longitudinal study, one can choose the number of persons to study and the frequency of measurement of each person. Options become even more involved for more complicated designs, such as those involving treatments at different levels. We illustrate here with examples of quick calculations for a survey and an experiment and then in Section 20.5 discuss a general approach for power calculations using simulations.

### *Standard deviation of the mean of clustered data*

Consider a survey in which it is desired to estimate the average value of $y$ in some population, and data are collected from $J$ equally sized clusters selected at random from a larger population, with $m$ units measured from each sampled cluster, so that the total sample size is $n = Jm$.[1] In this symmetric design, the estimate for the population total is simply the sample mean, $\bar{y}$. If the number of clusters in the population is large compared to $J$, and the number of units within each cluster is large compared to $m$, then the standard error of $\bar{y}$ is

$$\text{standard error of } \bar{y} = \sqrt{\sigma_y^2/n + \sigma_\alpha^2/J}. \tag{20.1}$$

[1] In the usual notation for survey sampling, one might use $a$ and $A$ for the number of clusters in the sample and population, respectively. Here we use the capital letter $J$ to indicate the number of selected clusters to be consistent with our general multilevel-modeling notation of $J$ for the number of groups in the data.

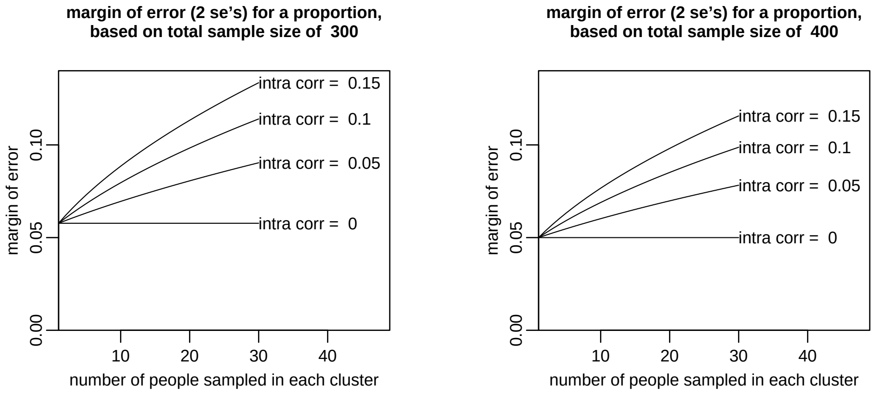


Figure 20.4 *Margin of error for inferences for a proportion as estimated from a cluster sample, as a function of cluster size and intraclass correlation, for two different proposed values of total sample size. The lines on the graphs do* not *represent a fitted model; they are based on analytical calculations using the variance formulas for cluster sampling.*

(The separate variance parameters $\sigma_y^2$ and $\sigma_\alpha^2$, needed for the power calculations, can be estimated from the cluster-sampled data using a multilevel model.)

This formula can also be rewritten as

$$\text{standard error of } \bar{y} = \sqrt{\frac{\sigma_{\text{total}}^2}{Jm}[1 + (m-1)\text{ICC}]}, \tag{20.2}$$

where $\sigma_{\text{total}}$ represents the standard deviation of all the data (mixing all the groups; thus $\sigma_{\text{total}}^2 = \sigma_y^2 + \sigma_\alpha^2$ for this simple model), and ICC is the *intraclass correlation*,

$$\text{intraclass correlation: ICC} = \frac{\sigma_\alpha^2}{\sigma_\alpha^2 + \sigma_y^2}, \tag{20.3}$$

the fraction of total variation in the data that is accounted for by between-group variation. The intraclass correlation can also be thought of as the correlation among units within the same group. Formulas (20.1) and (20.2) provide some intuition regarding the extent to which clustering can affect our standard errors. The greater the correlation among units within a group (that is, the bigger ICC is) the greater the impact on the standard error. If there is no intraclass correlation (that is, ICC = 0) the standard error of $\bar{y}$ is simply $\sigma_{\text{total}}/\sqrt{n}$.

### *Example of a sample size calculation for cluster sampling*

We illustrate sample size calculations for cluster sampling with a design for a proposed study of residents of New York City. The investigators were planning to study approximately 300 or 400 persons sampled for convenience from 10 or 20 U.S. Census tracts, and they wanted to get a sense of how much error the clustering was introducing into the estimation. The number of census tracts in the city and the population of each tract are large enough that (20.1) was a reasonable approximation.

Figure 20.4 shows the margin of error for $\bar{y}$ from this formula, as a function of the sample size within clusters, for several values of the intraclass correlation. When the correlation is zero, the clustering is irrelevant and the margin of error only depends on the total sample size, $n$. For positive values of intraclass correlation (so that people within a census tract are somewhat similar to each other, on average),

the standard error increases as the number of clusters decreases with fixed sample size. For the higher values of intraclass correlation shown in the graphs, it seems that it would be best to choose enough clusters so that no more than 20 persons are selected within each cluster.

But why, in Figure 20.4, do we think that interclass correlations between 0 and 15% are plausible? To start with, for binary data, the denominator of (20.3) can be reasonably approximated by 0.25 (since $p(1-p) \approx 0.25$ if $p$ is not too close to 0 or 1). Now suppose that the clusters themselves differ in some particular average outcome with a standard error of 0.2—this is a large value of $\sigma_\alpha$, with, for example, the percentages of Yes responses in some clusters as low as 0.3 and in others as high as 0.7. The resulting intraclass correlation is $0.2^2/0.25 = 0.16$. If, instead, $\sigma_\alpha = 0.1$ (so that, for example, the average percentage of Yes in clusters varies from approximately 0.4 to 0.6), the intraclass correlation is 0.04. Thus, it seems reasonable to consider correlations ranging from 0 to 5% to 15% as in Figure 20.4.

## 20.5 Multilevel power calculation using fake-data simulation

Figure 20.5a shows measurements of the immune system (CD4 percentage, transformed to the square root scale to better fit an additive model) taken over a two-year period on a set of HIV-positive children who were not given zinc. The observed noisy time series can be fitted reasonably well by a varying-intercept, varying-slope model of the form, $y_{jt} \sim \mathrm{N}(\alpha_j + \beta_j t,\, \sigma_y^2)$, where $j$ indexes children, $t$ indexes time, and the data variance represents a combination of measurement errors, short-term variation in CD4 levels, and departures from a linear trend within each child. This model can also be written more generally as $y_i \sim \mathrm{N}(\alpha_{j[i]} + \beta_{j[i]} t_i,\, \sigma_y^2)$, where $i$ indexes measurements taken at time $t_i$ on person $j[i]$. Here is the result of the quick model fit:

R output

```
lmer(formula = y ~ time + (1 + time | person))
            coef.est coef.se
(Intercept)  4.8      0.2
time        -0.5      0.1
Error terms:
 Groups   Name        Std.Dev. Corr
 person   (Intercept) 1.3
          time        0.7      0.1
 Residual             0.7
# of obs: 369, groups: person, 83
```

Of most interest are the time trends $\beta_j$, whose average is estimated at $-0.5$ with a standard deviation of 0.7 (we thus estimate that most, but not all, of the children have declining CD4 levels during this period). The above display also gives us estimates for the intercepts and the residual standard deviation.

We then fit the model in Bugs to get random simulations of all the parameters. The last three panels of Figure 20.5 show the results: the estimated trend line for each child, a random draw of the set of 83 trend lines, and a random replicated dataset (following the principles of Section 8.3) with measurements at the time points observed for the actual data. The replicated dataset looks generally like the actual data, suggesting that the linear-trend-plus-error model is a reasonable fit.

### *Modeling a hypothetical treatment effect*

We shall use these results to perform a power calculation for a proposed new study of dietary zinc. We would like the study to be large enough that the probability is

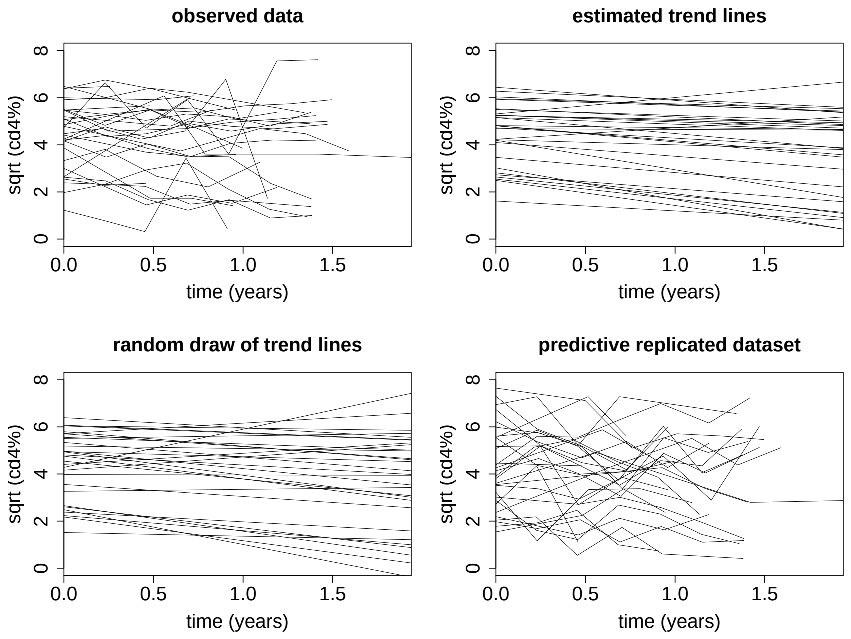


Figure 20.5 *(a) Progression of CD4 percentage over time (on the square root scale) for 83 untreated children j in the HIV study; (b) individual trend lines* $\hat{\alpha}_j + \hat{\beta}_j t$ *(posterior mean estimates from multilevel model); (c) a single posterior draw from the set of individual trend lines* $\alpha_j + \beta_j t$*; (d) a replicated dataset* $(\tilde{y}_{jt})$ *simulated from the posterior predictive distribution.*

at least 80% that the average estimated treatment effect is statistically significant at the 95% level.

*A hypothesized model of treatment effects.* To set up this power calculation we need to make assumptions about the true treatment effect and also specify all the other parameters that characterize the study. Our analysis of the HIV-positive children who did not receive zinc found an average decline in CD4 (on the square root scale) of 0.5 per year. We shall suppose in our power calculation that the true effect of the treatment is to reduce this average decline to zero.

We now set up a model for the hypothetical treatment and control data. So far, we have fitted a model to "controls," but that model can be used to motivate hypotheses for effects of treatments applied after the initial measurement ($t = 0$). To start with, the parameters $\alpha_j, \beta_j$ cleanly separate into an intercept that is unaffected by the treatment (and can thus be interpreted as an unobserved unit-level characteristic) and a slope $\beta_j$ that is potentially affected. A model of linear trends can then be written as

$$\begin{aligned} y_i &\sim \mathrm{N}(\alpha_{j[i]} + \beta_{j[i]} t_i,\, \sigma_y^2), \text{ for } i = 1, \ldots, n \\ \begin{pmatrix} \alpha_j \\ \beta_j \end{pmatrix} &\sim \mathrm{N}\left( \begin{pmatrix} \gamma_0^\alpha \\ \gamma_0^\beta + \gamma_1^\beta z_j \end{pmatrix}, \begin{pmatrix} \sigma_\alpha^2 & \rho\sigma_\alpha\sigma_\beta \\ \rho\sigma_\alpha\sigma_\beta & \sigma_\beta^2 \end{pmatrix} \right), \text{ for } j = 1, \ldots, J, \end{aligned}$$

where

$$z_j = \begin{cases} 1 & \text{if child } i \text{ received the treatment} \\ 0 & \text{otherwise.} \end{cases}$$

The treatment $z_j$ affects the slope $\beta_j$ but not the intercept $\alpha_j$ because the treatment can have no effect at time zero. As noted, we shall suppose $\gamma_0^\beta$, the slope for controls,

to be $-0.5$, with a treatment effect of $\gamma_1^\beta = 0.5$. We complete the model by setting the other parameters to their estimated values from the control data: $\mu_\alpha = 4.8$, $\sigma_\alpha = 1.3$, $\sigma_y = 0.7$, $\sigma_\beta = 0.7$. For simplicity, we shall set $\rho$, the correlation between intercepts and slopes, to zero, although it was estimated at 0.1 from the actual data.

*Design of the study.* The next step in the power analysis is to specify the design of the study. We shall assume that $J$ HIV-positive children will be randomly assigned into two treatments, with $J/2$ receiving regular care and $J/2$ receiving zinc supplements as well. We further assume that the children's CD4 percentages are measured every two months over a year (that is, seven measurements per child). We will now determine the $J$ required for 80% power, if the true treatment effect is 0.5, as assumed above.

### *Quick power calculation for classical regression*

We first consider a classical analysis, in which a separate linear regression is fitted for each child: $y_{jt} = \alpha_j + \beta_j t + \text{error}$. The trend estimates $\hat{\beta}_j$ would then be averaged for the children in the control and treatment groups, with the difference between the group mean trends being an estimated treatment effect. For simplicity, we assume the model is fitted separately for each child—that is, simple least squares, not a multilevel model.

This problem then has the structure of a simple classical sample size calculation, with the least squares estimate $\hat{\beta}_j$ being the single "data point" for each child $j$ and an assumed effect size $\Delta = 0.5$. We must merely estimate $\sigma$, the standard deviation of the $\hat{\beta}_j$'s within each group, and we can determine the required total sample size as $J = (2 \cdot 2.8\sigma/\Delta)^2$.

If $\hat{\beta}_j$ were a perfect estimate of the child's trend parameter, then $\sigma$ would simply be the standard deviation of the $\beta_j$'s, or 0.7 from the assumptions we have made. However, we must also add the variance of estimation, which in this case (from the formula for least squares estimation with a single predictor) is $\frac{1}{\sqrt{(-3/6)^2+(-2/6)^2+\cdots+(3/6)^2}}\sigma_y = 1.13\sigma_y = 0.8$ (based on the estimate of $\sigma_y = 0.7$ from our multilevel model earlier). The total standard deviation of $\hat{\beta}_j$ is then $\sqrt{\sigma_\beta^2 + 1.13^2\sigma_y^2} = \sqrt{0.7^2 + 0.8^2} = 1.1$. The sample size required for 80% power to find a statistically significant difference in trends between the two groups is then $J = (2 \cdot 2.8 \cdot 1.1/0.5)^2 = 150$ children total (that is, 75 per group).

This sample size calculation is based on the assumption that the treatment would, on average, eliminate the observed decline in CD4 percentage. If instead we were to hypothesize that the treatment would cut the decline in half, the required sample size would quadruple, to a total of 600 children.

### *Power calculation for multilevel estimate using fake-data simulation*

Power calculations for any model can be performed by simulation. This involves repeatedly simulating data from the hypothetical distribution that we expect our sampled data to come from (once we perform the intended study) and then fitting a multilevel model to each dataset. This can be computer-intensive, and practical compromises are sometimes needed so that the simulation can be performed in a reasonable time. Full simulation using Bugs is slow because it involves nested loops (100 or 1000 sets of fake data; for each, the looping of a Gibbs sampler required to

fit a model in Bugs). Instead, we fit the model to each fake dataset quickly using `lmer()`. We illustrate with the zinc treatment example.

*Simulating the hypothetical data.* The first step is to write a function in R that will generate data from the distribution assumed for the control children (based on our empirical evidence) and the distribution for the treated children (based on our assumptions about how their change in CD4 count might be different were they treated). This function generates data from a sample of $J$ children (half treated, half controls), each measured $K$ times during a 1-year period.

R code

```
CD4.fake <- function (J, K){
  time <- rep (seq(0,1,length=K), J)   # K measurements during the year
  person <- rep (1:J, each=K)          # person ID's
  treatment <- sample (rep (0:1, J/2))
  treatment1 <- treatment[person]
#                                      # hyperparameters:
  mu.a.true <- 4.8                     #   more generally, these could
  g.0.true <-  -.5                     #   be specified as additional
  g.1.true <-   .5                     #   arguments to the function
  sigma.y.true <-  .7
  sigma.a.true <- 1.3
  sigma.b.true <-  .7
#                                      # person-level parameters
  a.true <- rnorm (J, mu.a.true, sigma.a.true)
  b.true <- rnorm (J, g.0.true + g.1.true*treatment, sigma.b.true)
#                                      # data
  y <- rnorm (J*K, a.true[person] + b.true[person]*time, sigma.y.true)
  return (data.frame (y, time, person, treatment1))
}
```

The function returns a data frame with the simulated measurements along with the input variables needed to fit a model to the data and estimate the average treatment effect, $\gamma_1$. We save treatment as a data-level predictor (which we call `treatment1`) because this is how it must be entered into `lmer()`.

*Fitting the model and checking the power.* Next we can embed the fake-data simulation `CD4.fake()` in a loop to simulate 1000 sets of fake data; for each, we fit the model and obtain confidence intervals for the parameter of interest:

R code

```
CD4.power <- function (J, K, n.sims=1000){
  signif <- rep (NA, n.sims)
  for (s in 1:n.sims){
    fake <- CD4.fake (J, K)
    lme.power <- lmer (y ~ time + time:treatment1 +
      (1 + time | person), data=fake)
    theta.hat <- fixef(lme.power)["time:treatment1"]
    theta.se <- se.fixef(lme.power)["time:treatment1"]
    signif[s] <- (theta.hat - 2*theta.se) > 0     # returns TRUE or FALSE
  }
  power <- mean (signif)                          # proportion of TRUE
  return (power)
}
```

This function has several features that might need explaining:

- The function definition sets the number of simulations to the default value of 1000. So if `CD4.power()` is called without specifying the `n.sims` argument, it will automatically run 1000 simulations.

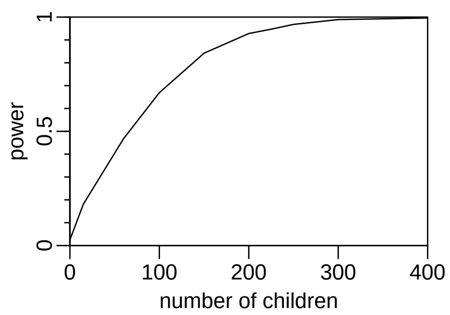


Figure 20.6 *Power (that is, the probability that estimated treatment effect is statistically significantly positive) as a function of number of children, $J$, for the hypothetical zinc study, as computed using fake-data simulation with multilevel inference performed by* `lmer()`. *The simulations are based on particular assumptions about the treatment effect and the variation among children and among measurements within children. We also have assumed $K = 7$ measurements for each child during the year of the study, a constraint determined by the practicalities of the experiment. Reading off the curve, 80% power is achieved at approximately $J = 130$.*

- The `lmer()` call includes the interaction `time:treatment1` and the main effect `time` but *not* the main effect `treatment1`. This allows the treatment to affect the slope but not the intercept, which is appropriate since the treatment is performed after time 0.
- The data frame `fake` is specified as an argument to `lmer()` so that the analysis knows what dataset to use.
- We assume the estimated treatment effect of the hypothetical study is statistically significantly positive if the lower bound of its 95% interval exceeds zero.
- The function returns the proportion of the 1000 simulations where the result is statistically significant; thus, the power (as computed via simulation) for a study with $J$ children measured at $K$ equally spaced times during the year.

*Putting it all together to compute power as a function of sample size.* Finally, we put the above simulation in a loop and compute the power at several different values of $J$, running from 20 to 400, and plot a curve displaying power as a function of sample size; the result is shown in Figure 20.6. Our quick estimate based on classical regression was that 80% power is achieved with $J = 150$ children (75 in each treatment group) also applies to the multilevel model in this case. The classical computation works in this case because the treatment is at the group level (in this example, persons are the groups, and CD4 measurements are the units) and the planned study is balanced.

At the two extremes:

- The power is 0.025 in the limit $J \to 0$. With a small enough sample, the treatment effect estimate is essentially random, and so there is a 2.5% chance that it is more than 2 standard errors above zero.
- Under the assumption that the true effect is positive, the power is 1 in the limit $J \to \infty$, at which point there are enough data to estimate the treatment effect perfectly.

Using simulation for power analyses allows for greater flexibility in study design. For instance, besides simply calculating how power changes as sample size increases, we might also have investigated a different kind of change in study design such as

changes in the percentage of study participants allocated to treatment versus control groups. This aspect of study design might be particularly relevant if treatment participants are more costly than control participants, for instance (see Exercise 20.6). Another design feature that could be varied is the number of measurements per person, and the simulation can also include missing data, nonlinearity, unequal variance, and other generalizations of the model.

## 20.6 Bibliographic note

Scott and Smith (1969), Cochran (1977), Goldstein and Silver (1989), and Lohr (1999) are standard and useful references for models used in survey sampling, and Groves et al. (2004) goes over the practical aspects of survey design. Montgomery (1986) and Box, Hunter, and Hunter (2005) review the statistical aspects of experimental design; Trochim (2001) is a more introductory treatment with useful practical advice on research methods.

Hoenig and Heisey (2001) and Lenth (2001) provide some general warnings and advice on sample size and power calculations. Design issues and power calculations for multilevel studies are discussed by Snijders and Bosker (1993), Raudenbush (1997), Snijders, Bosker, and Guldemond (1999), Raudenbush and Xiaofeng (2000), and Raudenbush and Bryk (2002).

## 20.7 Exercises

1. Sample size calculations for estimating proportions:
   (a) How large a sample survey would be required to estimate, to within a standard error of ±3%, the proportion of the U.S. population who support the death penalty?
   (b) About 14% of the U.S. population is Latino. How large would a national sample of Americans have to be in order to estimate, to within a standard error of ±3%, the proportion of Latinos in U.S. who support the death penalty?
   (c) How large would a national sample of Americans have to be in order to estimate, to within a standard error of ±1%, the proportion who are Latino?
2. Consider an election with two major candidates, A and B, and a minor candidate, C, who are believed to have support of approximately 45%, 35%, and 20% in the population. A poll is to be conducted with the goal of estimating the difference in support between candidates $A$ and $B$. How large a sample would you estimate is needed to estimate this difference to within a standard error of 5%? (Hint: consider an outcome variable that is coded as $+1$, $-1$, and 0 for supporters of A, B, and C, respectively.)
3. Effect size and sample size: consider a toxin that can be tested on animals at different doses. Suppose a typical exposure level for humans is 1 (in some units), and at this level the toxin is hypothesized to introduce a risk of 0.01% of death per person.
   (a) Consider different animal studies, each time assuming a linearity in the dose-response relation (that is, 0.01% risk of death per animal per unit of the toxin), with doses of 1, 100, and 10,000. At each of these exposure levels, what sample size is needed to have 80% power of detecting the effect?
   (b) This time assume that response is a logged function of dose and redo the calculations in (a).

4. Cluster sampling with equal-sized clusters: a survey is being planned with the goal of interviewing $n$ people in some number $J$ of clusters. For simplicity, assume simple random sampling of clusters and a simple random sample of size $n/J$ (appropriately rounded) within each sampled cluster.

   Consider inferences for the proportion of Yes responses in the population for some question of interest. The estimate will be simply the average response for the $n$ people in the sample. Suppose that the true proportion of Yes responses is not too far from 0.5 and that the standard deviation among the mean responses of clusters is 0.1.

   (a) Suppose the total sample size is $n = 1000$. What is the standard error for the sample average if $J = 1000$? What if $J = 100$, 10, 1?

   (b) Suppose the cost of the survey is \$50 per interview, plus \$500 per cluster. Further suppose that the goal is to estimate the proportion of Yes responses in the population with a standard error of no more than 2%. What values of $n$ and $J$ will achieve this at the lowest cost?

5. Simulation for power analysis: the folder `electric.company` contains data from the Electric Company experiment analyzed in Chapter 9. Suppose you wanted to perform a new experiment under similar conditions, but for simplicity just for second-graders, with the goal of having 80% power to find a statistically significant result (at the 95% level) in grade 2.

   (a) State clearly the assumptions you are making for your power calculations. (Hint: you can set the numerical values for these assumptions based on the analysis of the existing Electric Company data.)

   (b) Suppose that the new data will be analyzed by simply comparing the average scores for the treated classrooms to the average scores for the controls. How many classrooms would be needed for 80% power?

   (c) Repeat (b), but supposing that the new data will be analyzed by comparing the average gain scores for the treated classrooms to the average gain scores of the controls.

   (d) Repeat (b), but supposing that the new data will be analyzed by regression, controlling for pre-test scores as well as the treatment indicator.

6. Optimal design:

   (a) Suppose that the zinc study described in Section 20.5 would cost \$150 for each treated child and \$100 for each control. Under the assumptions given in that section, determine the number of control and treated children needed to attain 80% power at minimal total cost. You will need to set up a loop of simulations as illustrated for the example in the text. Assume that the number of measurements per child is fixed at $K = 7$ (that is, measuring every two months for a year).

   (b) Make a generalization of Figure 20.6 with several lines corresponding to different values of the design parameter $K$, the number of measurements for each child.
